# 3-M syndrome: evolution of the phenotype over time

**DOI:** 10.1186/s13052-025-02172-8

**Published:** 2025-12-23

**Authors:** Isabelle Bacchi, Sara Vandelli, Emanuele Coccia, Lucrezia Giannini, Roberta Zuntini, Rachele Teneggi, Stefano Giuseppe Caraffi, Maria Chiara Baroni, Gianluca Contrò, Adelaide Peruzzi, Irene Ambrosetti, Marzia Pollazzon, Chiara Sartori, Ekkehart Lausch, Uta Matysiak, Lucia Gambini, Giancarlo Gargano, Valeria Orlando, Antonio Novelli, Lorenzo Iughetti, Sheila Unger, Andrea Superti-Furga, Livia Garavelli

**Affiliations:** 1 Medical Genetics Unit, Azienda USL-IRCCS di Reggio Emilia, 42123 Reggio Emilia, Italy; 2 Department of Medical and Surgical Science (DIMEC), Alma Mater Studiorum University of Bologna, 40126 Bologna, Italy; 3 Pediatric Unit, Department of Medical and Surgical Sciences of the Mother, Children and Adults, University of Modena and Reggio Emilia, 41125 Modena, Italy; 4 Post-Graduate School of Pediatrics, University of Modena and Reggio Emilia, 41125 Modena, Italy; 5 Clinical Genetics Unit, Medical Direction, Azienda Ospedaliera Universitaria Integrata Verona, Verona, Italy; 6 Pediatric Unit, Azienda USL-IRCCS di Reggio Emilia, 42123 Reggio Emilia, Italy; 7 Pediatric Genetics, Center for Pediatric and Adolescent Medicine, University Hospital Freiburg, Freiburg, Germany; 8 Institute for Surgical Pathology, Medical Center, University of Freiburg, Freiburg, Germany; 9 Neonatal Intensive Care Unit, University Hospital of Parma, 43126 Parma, Italy; 10 Neonatal Intensive Care Unit, Azienda USL-IRCCS di Reggio Emilia, 42123 Reggio Emilia, Italy; 11 Translational Cytogenomics Research Unit, Laboratory of Medical Genetics, Bambino Gesu Pediatric Hospital, Roma, Lazio, Italy; 12 Division of Genetic Medicine, Lausanne University Hospital (CHUV) and University of Lausanne, Lausanne, 1011 Switzerland; 13 Genetica AG, Zurich and Lausanne, Lausanne, 1003 Switzerland

**Keywords:** 3 M syndrome, CUL7, *OBSL1* and *CCDC8*

## Abstract

**Background:**

3-M syndrome is an autosomal recessive disease characterized by short stature, facial dysmorphism and skeletal anomalies. To date, biallelic pathogenic *CUL7* variants are responsible for the majority of cases, but biallelic deleterious changes in *OBSL1* and *CCDC8* can also establish the diagnosis.

**Cases presentation:**

We report two unrelated newborns showing clinical signs compatible with 3-M syndrome and we describe the evolution of the phenotype of the first patient over time. Molecular analysis identified two compound heterozygous *CUL7* variants in the first individual and a homozygous *CUL7* variant in the second one.

**Conclusions:**

We reviewed the literature highlighting the clinical differences between patients with variants in *CUL7*,* OBSL1* and *CCDC8.* Our paper highlights how the clinical diagnosis of 3-M is easier in the first months of life, while in older children the phenotype becomes increasingly nuanced. It also underlines the clinical relevance of Next Generation Sequencing and functional studies, which may be necessary to confirm the pathogenicity of some variants, becoming an essential part of the multidisciplinary management of patients.

**Supplementary Information:**

The online version contains supplementary material available at 10.1186/s13052-025-02172-8.

## Background

3-M syndrome (OMIM# 273750, 612921, 614205) is a rare autosomal recessive disease characterized by severe pre- and post-natal growth retardation, bulbous nose, short thorax, prominent abdomen, slight slender tubular bones, tall vertebral bodies, and normal intelligence. It was first described in 1975 by Miller, McKusick and Malvaux, hence the name “3M” [1]. Biallelic pathogenic variants in the *CUL7* gene, first described in 2005, are responsible for the majority of cases (OMIM #273750) [2]. Biallelic pathogenic variants in *OBSL1* (OMIM #612921) and *CCDC8* (OMIM #614205), identified in 2009 and 2011 respectively [3, 4], can also establish the diagnosis. More than 200 patients with molecular confirmation have been described to date [2–41].

*CUL7* encodes a protein called cullin 7, involved in the E3 ubiquitin-protein ligase complex [42]. E3 ubiquitin ligases catalyze the ubiquitination process, i.e. the binding of ubiquitin proteins to specific protein substrates, that are subsequently targeted for degradation and recycling via the proteasome complex [2, 6, 43]. E3 ligases are crucial in physiological bone growth and bone metabolism [44]. The vast majority of pathogenic and likely pathogenic variants are frameshift and nonsense variants, followed by splice site and missense variants. Small deletions are prevalent over small duplications.

*OBSL1* codes for the obscurin-like protein 1, a cytoskeletal adaptor and a regulator of the Cul7-RING (FBXW8) ubiquitin-protein ligase. Deleterious variants in the *OBSL1* gene are mainly frameshift and nonsense variants, but rare splice site and missense variants have also been reported in the literature. Small duplications are slightly more represented than deletions [3, 8].

*CCDC8* encodes the coiled-coil domain containing protein 8, a cofactor required for p53-mediated apoptosis following DNA damage. It does not interact directly with cullin 7, but coimmunoprecipitation studies indicate a physical interaction with obscurin-like protein 1 [44]. To our knowledge, deleterious variants identified in *CCDC8* are usually missense, while duplications and delins are very rare [11, 42].

Here we review and re-evaluate a comprehensive selection of clinical information from all the individuals affected by 3 M syndrome described in the literature to date. Moreover, we present two novel cases with biallelic variants in the *CUL7* gene, and we describe their clinical features typical of 3 M syndrome over time.

## Methods

### Genetic analysis

#### Patient 1

DNA was extracted from peripheral blood samples of the proband and his parents.

Single nucleotide polymorphism array (SNP-array) was performed using Illumina CytoSNP-850 K with an average coverage of 100Kb and an average spacing of 5Kb-1Kb in the relevant gene regions, following the guidelines of the International Collaboration for Clinical Genomics (ICCG) and the Cancer Cycogenomics Microarray Consortium (CCMC).

-Trio Whole Exome Sequencing (WES) analysis was performed using ClinEX pro kit (4bases) on Illumina NovaSeq6000 platform. Only coding regions and exon-intron junctions (± 5 bp) of genes associated with skeletal dysplasias where studied. Analytical sensitivity and specificity were higher than 99%. The average coverage of the aligned regions was 278.63X. Only regions with a minimum read depth of 30x were considered for interpretation.

For transcript analysis, total RNA was isolated from peripheral blood mononuclear cells, using RNeasy Mini Kit (Qiagen). A total of 1 µg RNA was reverse-transcribed into cDNA using Oligo-dT and Random Hexamer primers using Transcriptor First Strand cDNA synthesis kit (Roche). PCR for the evaluation of splicing alteration was performed with specific forward primers (TCAACTGCCATGTCTACAAG) and specific reverse primers (ATTCAGCACCACGGCATAG) under standard condition. PCR products were analyzed by standard gel electrophoresis and sequenced on an ABI 3500 Genetic Analyzer (Thermo Fisher Scientific). β-actin was used as internal control.

All the variants were annotated using NCBI transcripts (GRCh38), and classified according to the American College of Medical Genetics and Genomics and the Association for Molecular Pathology (ACMG/AMP) recommendations [45, 46].

#### Patient 2

DNA was extracted from peripheral blood samples of the proband and his parents.

Whole exome sequencing (WES) was performed using the Ion AmpliSeq™ Exome RDY Kit on the Ion Torrent S5 Sequencer, AmpliSeq Exome Hi-Q analysis workflow (Germline) − 540 - w1.2 – Single Sample r.0. The non-coding intronic regions that are analyzed for genes extend for approximately 20 nucleotides upstream and downstream of each exon. The sequences are aligned to the Genome Reference Consortium Human Build 37 (GRCh37) reference genome. Confirmatory analysis of pathogenic (C5), likely pathogenic (C4) or of uncertain clinical significance (C3) variants was performed by Automated Direct Sequencing on the 3500 Dx.

Genetic Analyzer (Thermo Fisher Scientific). Average coverage of sequenced regions 100X.

### Review of the literature

The aim of this review was to analyze the state of the art of the clinical phenotype of patients with 3-M Syndrome. We searched MEDLINE (PubMed) with the keywords ”3 M syndrome”, ”3- M syndrome”, “THREE M syndrome” and using the filter “Human”, limited to articles written in English and published up to March 2025 to ensure optimal data collection. In order to standardize our analysis, we excluded cases without a molecular diagnosis and reports with insufficient clinical information. Only post-natal cases were included.

We retrieved 36 publications, from 1975 to March 2025, describing a total of 217 individuals with genetic confirmation of 3-M syndrome; 177 of these included X-ray imaging data.

We collected information about gender and age, anthropometric parameters at birth and at the specified evaluation, clinical and radiological features, neurodevelopment, and genetic characteristics. The collected information from each article is shown in Supplementary Tables 1–3. Not all the features were assessed in all patients. Some case reports in the literature are more detailed than others, so we summarized the available data evaluated by the different authors. Where photographs of patients were available, we inferred some missing information from the photographs.

### Statistical analysis

Given the limited number of samples and qualitative nature of clinical feature assessment, statistical analysis was mainly descriptive. The proportion of each variant in the male and female population was assessed using a binomial test, with a confidence interval (CI) of 95%. The Comparisons between frequencies were performed using Chi-square test or Fisher’s exact test as appropriate, depending on sample size, with a CI of 95%.

## Clinical report

### Patient 1

#### First evaluation at 6 months

The first male patient initially came to our attention at the age of 6 months. The family history revealed a maternal lineage predisposition to short stature. At birth, length was 41 cm (< 1st centile, -3.42 SD), weight was 2,010 g (1st centile, -2.48 SD) and head circumference was 33 cm (10th centile, -1.27 SD). At the age of 6 months, the child had a length of 58 cm (< 1st centile, -3.70 SD) and a weight of 5,250 g (< 1st centile − 3.14 SD). The head circumference was 43.5 cm (45th centile, -0.12 SD). Pubertal stages were A0P1B1. Psychomotor development was appropriate for the age.

**Fig. 1:**
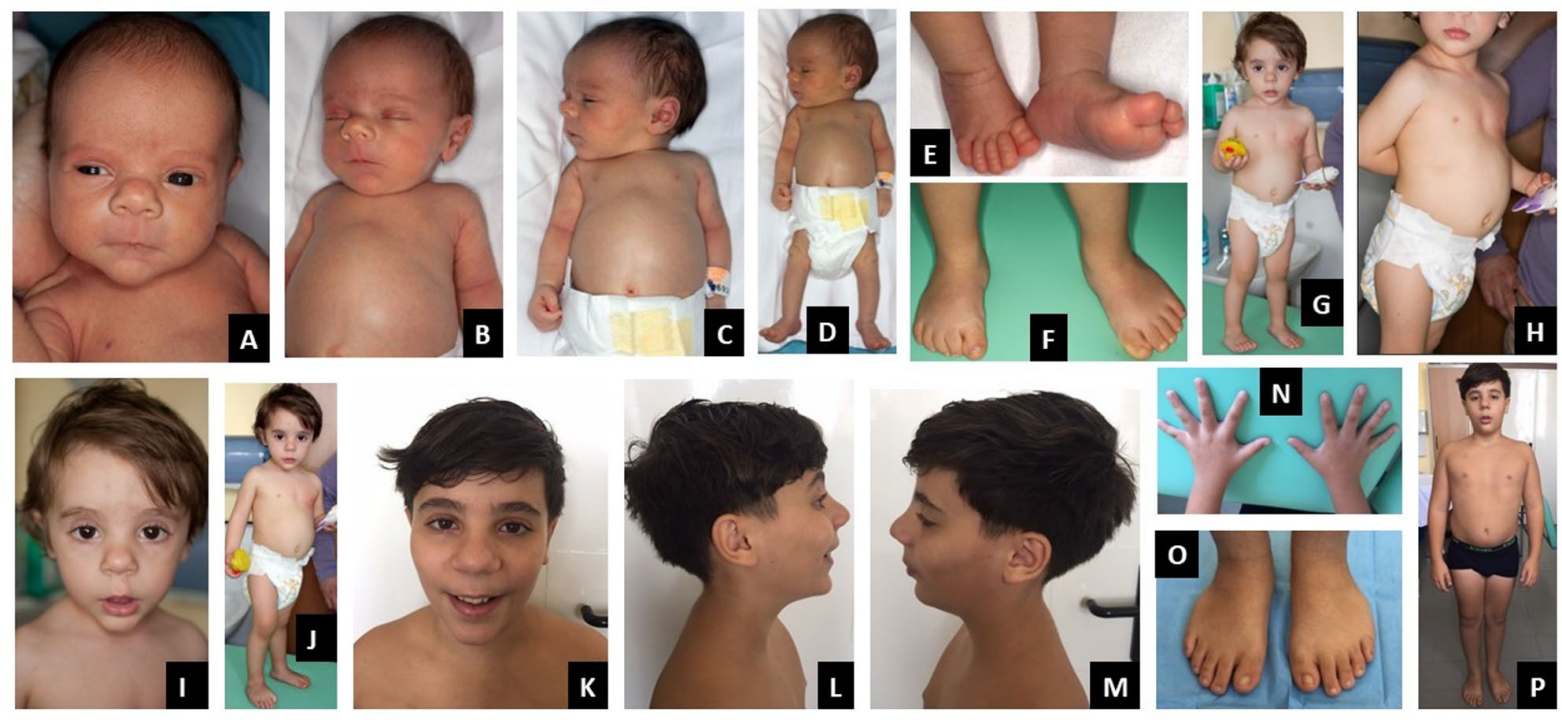
Phenotype of patient 1 at the age of 6 months, 2 years and 12 years. Phenotype at the age of 6 months: relative macrocephaly, dolichocephaly, short neck, squared off shoulders, pectus excavatum, short and broad thorax, protuberant abdomen with diastasis recti, short limbs and prominent calcaneus. Main facial features: triangular face, frontal bossing, hypoplastic midface, full eyebrows, bulbous nose, long philtrum, pointed chin, protruding ears with the impression of a melancholic aspect of the face. (**A**-**E**). Phenotype at the age of 2 years: squared off shoulders, protuberant abdomen and prominent calcaneus. Main facial features: triangular face, thick eyebrows, bulbous nose, long philtrum, pointed chin (**F**-**J**). Phenotype at the age of 12 years: Rather short thorax, slightly protuberant abdomen with diastasis recti. Main facial features: slightly depressed nasal bridge, bulbous nasal tip pointing upwards, slightly pointed chin, bilateral brachydactyly (**K**-**P**)

At the physical examination, the patient exhibited relative macrocephaly, dolichocephaly, and frontal bossing. He had short neck with prominent trapezius muscles, squared off shoulders, winged scapulae, pectus excavatum, short and broad thorax, hyperlordosis, protuberant abdomen with diastasis recti, short limbs and prominent calcaneus. He showed hypotonia and joint laxity. The main facial features included triangular face, hypoplastic midface, full eyebrows, bulbous nose, long philtrum, pointed chin, protruding ears – overall, the face gave the impression of a melancholic aspect (Fig. 1A-E).

**Fig. 2:**
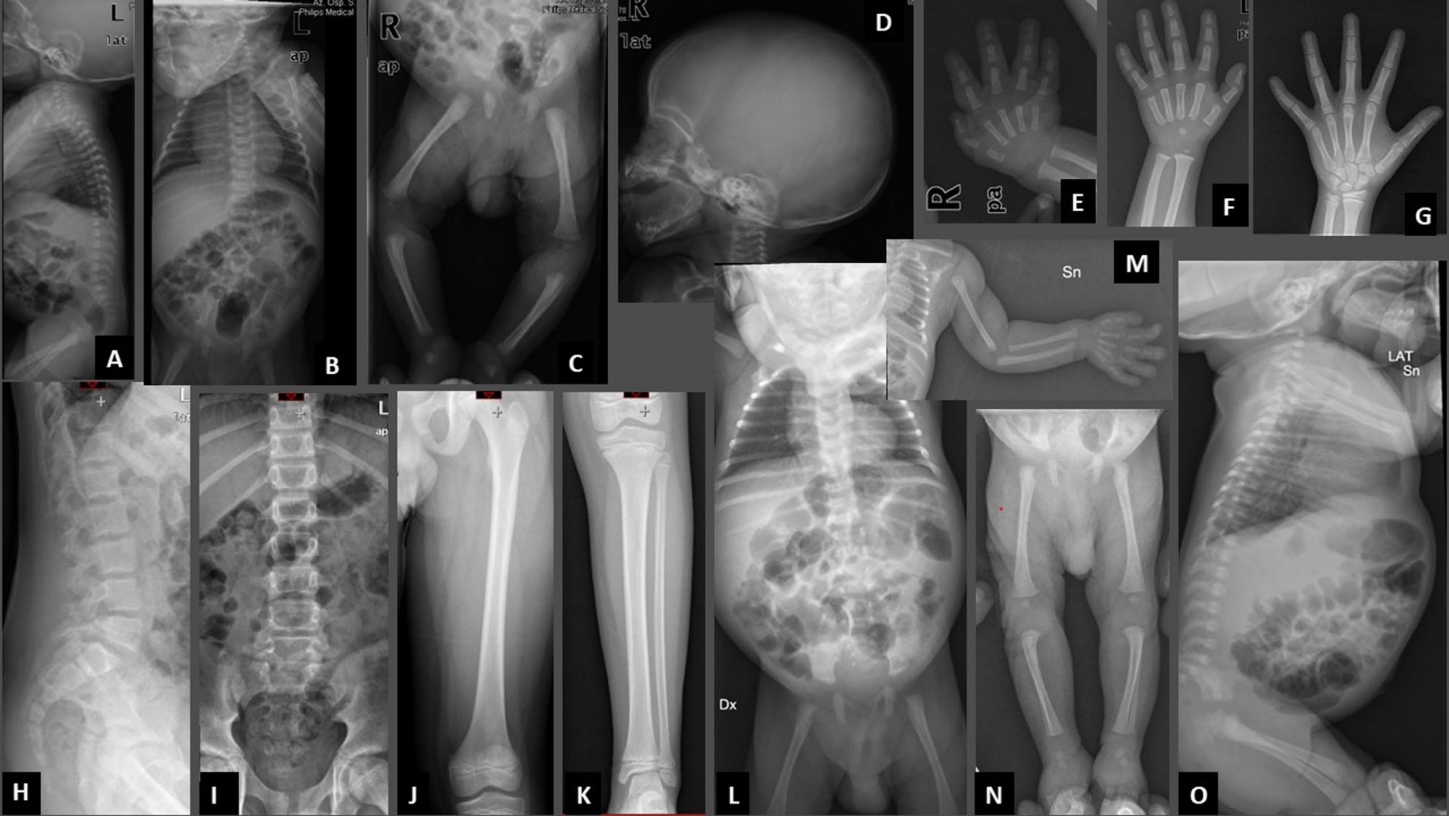
Radiographs of patients 1 and 2. X-rays of patient 1 at birth: slender and elongated long bones, with diaphyseal constriction and cortical thickening (**C**); tall vertebrae, with reduced anteroposterior diameter (**A**, **B**); thin metacarpals and phalanges (**E**). Left wrist and hand X-ray of patient 1 at the age of 2 years: Skeletal maturation assessment on X-rays of the left wrist and hand with the Greulich and Pyle method: marked delay, with bone age of 9 months at the chronological age of 2 years (**F**). X-rays of patient 1 at the age of 12 years: delayed skeletal maturation, with a bone age of 9–10 years. Slightly tall lumbar vertebral bodies, thin long bones (**G**-**K**). X-rays of patient 2 at birth: slightly slender bones and lightly flared metaphyses of the distal femur and proximal tibia without other evident signs of skeletal dysplasias (**L**-**O**)

X-ray showed slender and elongated long bones, with diaphyseal constriction and cortical thickening (Fig. 2C); the vertebrae appeared tall with reduced anteroposterior diameter (Fig. 2A, B); the bones of both hands were thin (Fig. 2E).

#### Evaluation at 2 years

The child was next evaluated at the age of two years. His height was 77 cm (< 1st centile, -2.92 SD), weight was 8,150 g (< 1st centile, -3.71 SD), and head circumference was 50 cm (83rd centile, + 0.96 SD). Pubertal stages were A0P1B1, and testicular volume was 1 cc bilaterally. On physical examination, his shoulders appeared squared off, the protuberant abdomen and prominent calcaneus were still evident. The main facial features were unchanged: triangular face, thick eyebrows, bulbous nose, long philtrum, pointed chin (Fig. 1F-J).

Skeletal maturation assessment on X-rays of the left wrist and hand with the Greulich and Pyle method showed a marked delay, with bone age of 9 months at the chronological age of 2 years (Fig. 2F).

#### Evaluation at 12 years

The patient was unavailable to follow-up for about 10 years, until he was referred for genetic counseling again because of concerns about poor growth. At the age of 12 years, his biometric parameters were: height 136.3 cm (4th centile, -1.76 SD), weight 42.5 Kg (57th centile, + 0.17 SD) and head circumference 55.5 cm (88th centile, + 1.19 SD). Puberal stages were A + P3B1 and testicular volume was 5 cc bilaterally. Seated height was 73.5 cm (3rd centile), span 139 cm, and seated height/height ratio 0.54 (+ 2.5 SD). He was well below his genetic target of 179.5 cm. On physical examination, he had slightly depressed nasal bridge, bulbous nasal tip pointing upwards, slightly pointed chin, bilateral brachydactyly, and single transverse palmar crease on the right hand. The thorax was rather short, but the hint of pectus excavatum that was present as a child was no longer evident. The examination showed a slightly protuberant abdomen with diastasis recti. Heels were no longer prominent. Bilateral flat foot was noticed as well as joint laxity, especially distal. Phenotype was otherwise normal, with regular body proportions (Fig. 1K-P).

The radiological evaluation showed delayed skeletal maturation, with a bone age of 9–10 years. The lumbar vertebral bodies appeared slightly tall and the long bones appeared thin (Fig. 2G-K).

#### Genetic testing

Chromosomal microarray analysis, by way of SNP-array analysis, identified no copy number variants or Loss of Heterozygosity regions.

WES performed on the proband-parents trio and filtered for genes associated with skeletal dysplasias revealed two compound heterozygous variants in the *CUL7* gene (NM_014780.5): a missense variant inherited from his mother, c.4391 A > C p.(His1464Pro), already reported in the literature and classified as likely pathogenic (Huber_2005, Li_2014, Yan_2014), and an intronic variant of uncertain significance (VUS) inherited from his father, c.2063 + 5G > C p.(?).

**Fig. 3:**
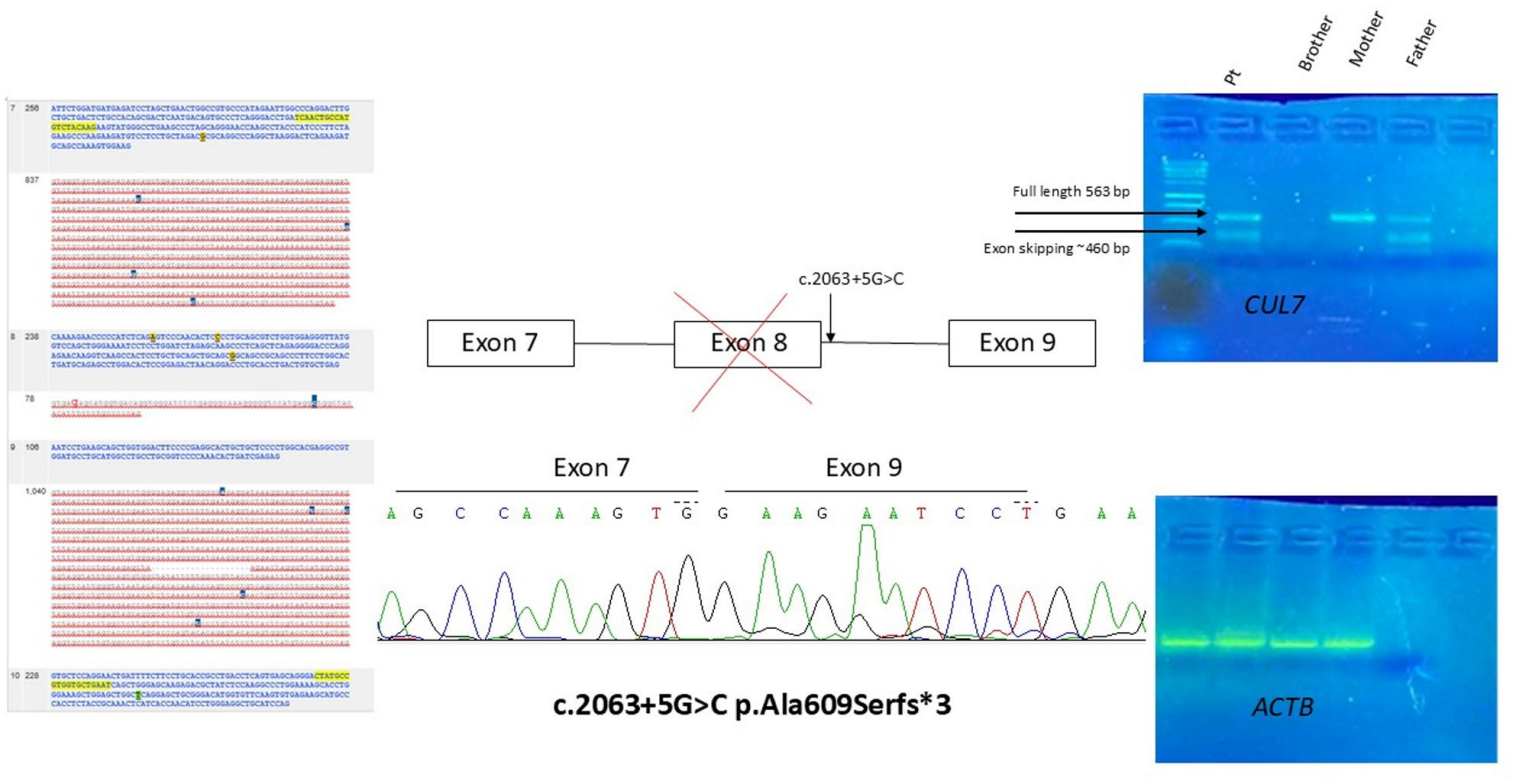
Functional analysis. Transcript analysis on mRNA from the patient’s peripheral blood performed to assess the causative role of the c.2063 + 5G > C variant showed that it is responsible for skipping of exon 8, causing the formation of a premature stop codon, p.Ala609Serfs*3

This VUS is extremely rare in population databases (gnomAD v4.1.0, accessed on 18/04/2025) and in silico predictions (SpliceAI) suggest a possible effect on splicing. Transcript analysis on mRNA from the patient’s peripheral blood performed to assess the causative role of the c.2063 + 5G > C variant showed that it is responsible for skipping of exon 8, causing the formation of a premature stop codon, p.Ala609Serfs*3 (Fig. 3). This functional study allowed us to reclassify the variant as likely pathogenic and confirmed the diagnosis of 3 M syndrome.

### Patient 2

#### Evaluation at 4 months

**Fig. 4:**
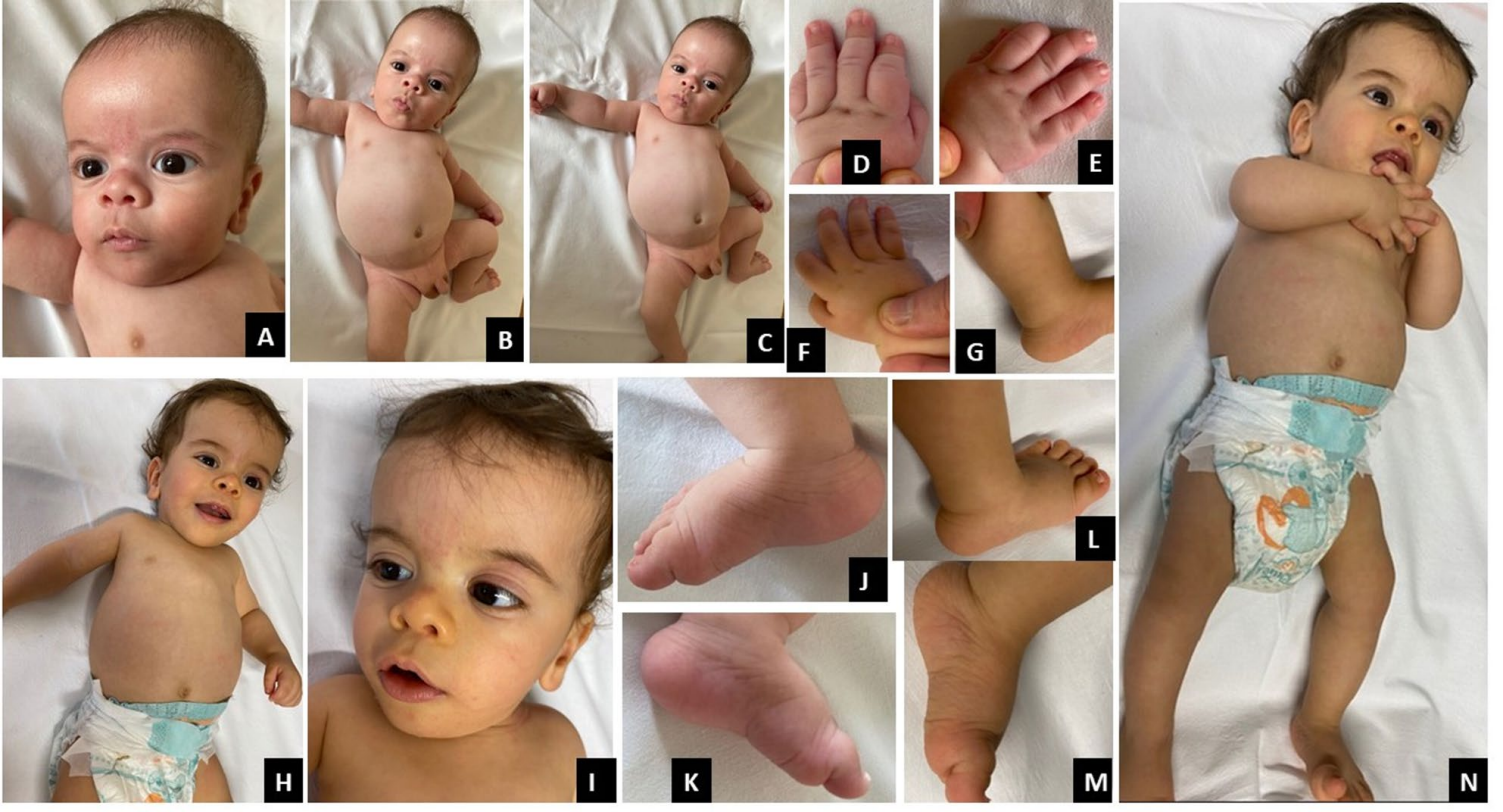
Phenotype of patient 2 at the age of 4 months and 9 months. Phenotype at the age of 4 months: relative macrocephaly, very short thorax, very protuberant abdomen. Main facial features: high and rounded forehead, slight frontal bossing, depressed nasal bridge, bulbous nasal tip, (**A**-**C**), brachydactyly, (**D**-**F**), prominent calcaneus (**G**). Phenotype at the age of 9 months: relative macrocephaly, short thorax, protuberant abdomen. Main facial features: high forehead, slight frontal bossing, depressed nasal bridge, bulbous nasal tip, (**H**-1, **N**) prominent calcaneus (**J**-**M**)

The second male patient came to us for evaluation at the age of 4 months. The parents were first cousins and the child had three healthy siblings. During pregnancy, ultrasound indicated the possibility of a skeletal dysplasia, but the mother was unwilling to undergo prenatal invasive investigations. The child was born at 40th week of gestation with a birth weight of 2,200 g (1st centile, -2.18 SD), length of 41 cm (< 1st centile, -3.42 SD) and head circumference of 34 cm (20th centile, -0.83 SD). Apgar score was 9 at 1 and 5 min. The patient was admitted to the Neonatal Department where high-flow oxygen therapy was administered for 2 days. During hospitalization a right inguinal hernia was diagnosed. The babygram x-rays showed slightly slender bones and lightly flared metaphyses of the distal femur and proximal tibia without other evident signs of skeletal dysplasias (Fig. 2L-O). Transfontanellar ultrasound, brain MRI, echocardiography, auditory evoked potentials and abdominal ultrasound were all normal. At the age of 4 months, he presented the following measurements: length 52 cm (< 1st centile, -4.75 SD), weight 3,950 g (< 1st centile, -3.59 SD), head circumference 40 cm (7th centile, -1.46 SD); his genetic target for height was 175.5 cm. Pubertal stages were A0P1B1 and testicular volume was 1–2 ml bilaterally. He had relative macrocephaly, a high and rounded forehead, a slight frontal bossing, depressed nasal bridge, bulbous nasal tip, very short thorax, very protuberant abdomen (Fig. 4A-C), brachydactyly of both hands (Fig. 4D-E), prominent calcaneus (Fig. 4J-K), mild hypotonia, no limitation of elbow extension.

#### Evaluation at 9 months

At the last evaluation at the age of 9 months his auxological parameters were: length 57 cm ( < < 3rd centile, -5.37 SD), weight 4,940 g ( < < 3rd centile, -4.48 SD), head circumference 43 cm (4th centile, -1.74 SD) Pubertal Stages were A0P1B1 with testicular volume 1–2 ml and mobile right testicle. He still had right a inguinal hernia (Fig. 4F-I; L-N).

#### Genetic testing

WES analysis showed the deletion of six nucleotides in *CUL7*, the last four of intron 4 and the first two of exon 5, c.1234-4_1235del in the homozygous state inherited from both parents. This alteration causes the loss of the canonical slicing acceptor site and a portion of exon 5 leading to an alteration of the transcript and consequently of the synthesis of the protein. The variant is not present in gnomAD v.4.1.0 (accessed on 18/04/2025) and was classified as likely pathogenic.

## Discussion

When faced with a newborn or infant with a frankly pathological length as in our two cases, it is natural for the neonatologist and the pediatrician to think of a skeletal dysplasia and request a radiological examination such as an antero-posterior and latero-lateral babygram. In the case of 3-M syndrome, it is surprising how the radiological evaluation can often detect signs that are too vague to clearly orient the diagnostic process. If a precise suspicion has not already been formulated by the neonatologist, the pediatrician or the clinical geneticist, these signs can easily be overlooked. For this reason, it is of fundamental importance to define the most significant clinical and radiological signs in newborns and infants.

In reviewing the literature, we retrieved 217 cases of 3-M syndrome with molecular diagnosis fitting the inclusion criteria: 135 with *CUL7*, 70 with *OBSL1*, and 12 with *CCDC8* variants. Among cases with variants in the *CUL7* gene, about one third (43/135) belong to a population isolated with high genetic homogeneity, the Yakuts, described by Maksimova et al. in 2007 [5]. 3-M syndrome cases from Yakut families share the same pathogenic variant, NM_014780.5:c.4581dup (also reported as c.4582insT in previous works), and were noted to have some distinctive features, in particular a lower occurrence of radiological findings. We propose that including the Yakut population may lead to a bias in the generalized clinical comparison of *CUL7*-related 3-M syndrome patients, given that the remaining cases are 92 individuals from 62 independent families, encompassing 62 different variants. For these reasons, in our review we considered the Yakut population separately, and we excluded them while calculating the total frequency of each clinical feature. Indeed, comparing 3-M syndrome cases in the Yakut population with all other *CUL7*-related cases, we confirmed that the radiological findings are significantly less frequent in the Yakuts (Supplementary Table 5): these include slender long bones (79% vs. 2%, *P* = 2.2E-08), tall vertebral bodies (63% vs. 9%, *P* = 6.6E-05) and flared metaphyses (91% vs. 26%, *P* = 0.003). Another significant difference concerns clinodactyly (62% vs. 9%, *P* = 7.7E-05), while all other aspects of 3 M syndrome are comparable between the two groups. For ease of reference with previous articles [41], in the Supplementary Materials we also provide the frequencies of each feature including the Yakut population (Supplementary Table 6). As expected, in this total the prevalence of the radiological features is markedly lower. This may lead to underestimating the central role of radiological evaluation in the differential diagnosis of 3 M syndrome from other short stature syndromes.

In the supplementary materials, we provide tables schematizing the clinical features and variants collected (*CUL7*: Supplementary Tables 1 and 6, *OBSL1*: Supplementary Table 2,* CCDC8*: Supplementary Table 3). A comparison of the prevalence of the clinical characteristics of 3 M syndrome patients, divided according to the gene involved, is provided in Table 1 and Supplementary Table 4.

**Table 1:** Comparison of phenotypic characteristics in patients with variants in *CUL7*, *OBSL1* and *CCDC8*

	Individual with CUL7 diagnosis		Individual with OBSL1 diagnosis		Individual with CCDC8 diagnosis	
**N** **(n per gender)**	92 (34 F/58 M)	53%	70 (34 F/36 M)	40%	12 (6 F/6 M)	7%
**Birth weight in SD**	-3.02 ± 1.03 (-2.90)		-2.69 ± 0.81 (-2.71)		-3.43 ± 1.52 (-3.05)	
**Parameters at first reporting**						
Age in years	6.04 ± 6.81 (3.00)		7.05 ± 9.37 (4.50)		4.27 ± 4.00 (3.40)	
Weight in SD	-3.78 ± 1.73 (-4.0)		-3.26 ± 1.80 (-3.39)		-3.65 ± 1.23 (-3.9)	
Height in SD	-5.32 ± 1.32 (-5.20)		-4.49 ± 1.45 (-4.60)		-4.34 ± 1.64 (-3.5)	
Head circumference in SD	-0.75 ± 1.32 (-0.77)		-0.75 ± 1.55 (-0.81)		-1.56 ± 1.13 (-1.0)	
**Facial features**						
Dolichocephaly	50/79	63%	39/58	67%	3/12	25%
Triangular face	82/89	92%	54/62	87%	6/12	50%
Frontal bossing	76/90	84%	46/59	78%	11/12	92%
Hypoplastic midface	73/82	89%	41/59	69%	6/12	50%
Bulbous nose	84/90	93%	56/62	90%	8/12	67%
Anteverted nares	58/63	92%	35/44	80%	8/12	67%
Long philtrum	77/83	93%	47/58	81%	4/10	40%
Full lips	72/85	85%	45/60	75%	6/12	50%
Pointed chin	75/84	89%	50/62	81%	7/10	70%
Prominent ears	35/53	66%	23/37	62%	5/9	56%
Midline facial haemangioma	11/37	30%	6/32	19%	2/5	40%
Lower eyelid fat pads	18/54	33%	13/41	32%	0/6	0%
Thick eyebrows	50/67	75%	36/45	80%	4/6	67%
Prominent central tubercle of upper lip	21/37	57%	17/23	74%	2/5	40%
**Other features**						
Short neck	76/84	90%	45/58	78%	6/10	60%
Short thorax	68/72	94%	42/50	84%	7/8	88%
Pectus deformity	36/66	55%	28/53	53%	2/10	20%
Squared-off shoulders	28/35	80%	16/23	70%	3/7	43%
Winged scapulae	8/18	44%	2/12	17%	0/7	0%
Chest groove	14/27	52%	12/25	48%	2/7	29%
Protuberant abdomen	30/35	86%	22/27	81%	0/1	NA
**Spine**						
Hyperlordosis	52/72	72%	36/51	71%	7/12	58%
Scoliosis	11/62	18%	13/48	27%	2/12	17%
**Extremities**						
Hip dislocation	12/62	19%	1/29	3%	2/10	20%
Joint hypermobility	42/59	71%	22/47	47%	4/12	33%
Clinodactyly	47/76	62%	32/55	58%	3/12	25%
Unique transverse palmar crease	14/22	64%	2/9	22%	0/0	NA
Pes planus	49/56	88%	31/39	79%	0/1	NA
Spina bifida occulta	8/36	22%	1/25	4%	1/11	9%
Prominent heels	73/81	90%	40/50	80%	9/12	75%
**Radiological features**						
Slender long bones	57/72	79%	41/51	80%	7/11	64%
Tall vertebral bodies	45/71	63%	33/50	66%	5/11	45%
Flared metaphyses	30/33	91%	17/23	74%	1/1	NA
Diaphyseal constriction	37/43	86%	24/28	86%	1/1	NA
**Neurodevelopment**						
**Psychomotor delay**	8/45	18%	5/25	20%	1/5	20%
Normal Intellect	39/39	100%	20/22	91%	5/5	100%

In the table some clinical features are derived from pictures of articles. List of clinical features was adapted from Hanson et al., 2009 and Lugli et al., 2016. Birth weight, age, height and weight at the presentation are reported as mean ± standard deviation (median). F, female; M, male; SD, standard deviation; NA: not available; 0/0 if this characteristic has not been assessed in that patient and cannot be deduced from the photographic images

Variants in *CUL7* were reported more frequently in males (63%, p value 0.016), while variants in *OBLS1* and *CCDC8* have almost overlapping frequency in males and females. The mean age at reporting was higher in *OBSL1* patients (7.05 ± 9.37 years) than in *CUL7* (6.04 ± 6.81 years) or *CCDC8* patients (4.27 ± 4.00 years). Nearly all patients displayed short stature at the time of first reporting, with average height slightly lower in *CUL7* patients (Table 1). Interestingly, only two patients with *OBSL1*-related 3 M syndrome had normal height [24, 37], while all others had borderline (3rd centile, -1.9 SD) to severe short stature (< 1st centile, up to -9.3 SD).). On average, the head circumference at the presentation was in the low range of normal in *CCDC8* patients (-1.56 ± 1.13 SDS), whereas it was close to normal in the other patients, with a few outliers.

Overall, when considering the phenotype of 3 M syndrome as a whole, we often think of the *CUL7*-related phenotype, because of the earlier identification and the greater number of cases.

Based on the observations in our patients (Figs. 1A, B, C, D and E and 4A, B, C, J and K) and from the review of the literature, including an accurate re-examination of photographs from published cases, we propose that the main features for guiding clinical suspicion, already evident in the neonatal period, are short length/short stature, short thorax, protuberant abdomen, prominent heels, and a characteristic appearance of the nose, with a bulbous or fleshy nasal tip, anteverted nares and often a depressed, concave bridge. These features are well represented in all three gene-related categories, although for *CCDC8* it should be noted that the number of patients reported to date is very small and comparisons are not currently reliable. We also highlight the mild radiological signs (Fig. 2) such as slender long bones, tall vertebral bodies and flared metaphyses, which must be carefully evaluated together with radiologists expert in skeletal dysplasias, because they are very mild, but significant nonetheless, as it is our task to establish a differential diagnosis between the various and numerous skeletal dysplasias. In the two novel patients described here, all these characteristic clinical features of 3-M syndrome were present from the first months of life, and guided the genetic investigations.

At later ages, it is much more difficult to suspect 3-M syndrome because the radiological signs are always vague and the clinical signs are less and less evident: in addition to the short stature, there remains a slightly protuberant abdomen that may be overlooked by the clinician because it looks like a common anatomical variant, and the characteristic appearance of the nose. The latter must be taken into consideration because it is truly particular and typical of this condition.

Due to their relevance in the follow-up care, it is also important to consider the skeletal features, such as pectus deformity, winged scapulae, hyperlordosis, scoliosis, hip dislocation, joint hypermobility, pes planus and spina bifida occulta. Scoliosis is more frequent in patients with variants in *OBSL1* (27% of cases, compared with 18% of patients with variants in *CUL7* and 17% of patients with variants in *CCD8*), while all the others are slightly more represented in patients with variants in *CUL7*. It is therefore important in these patients to have a follow-up care with periodic pediatric orthopedic and physiatric check-ups.

About 20% of the patients, evenly distributed among the three variants, showed psychomotor development delay, despite normal intelligence in adulthood. It should be noted that only patients with variants in *OBSL1* presented with intellectual disability, while the majority of patients with *CUL7*- or *CCDC8*-related 3-M syndrome and available neuropsychological assessment data had normal intellect. *OBSL1* is known to play an important role in the ubiquitin ligase pathway that regulates Golgi morphogenesis and dendrite patterning in the brain [47].

## Conclusions

The diagnosis of 3-M syndrome is usually quite evident in the first months of life, while it can be much more difficult at later ages. In particular, in a newborn, in the presence of reduced length, the most important clinical features are the bulbous tip of the nose, the short thorax, the protuberant abdomen and the prominent calcaneus. Radiological signs are generally very vague in the newborn, but a careful evaluation can highlight the presence of thin long bones, flared metaphyses and slightly tall vertebral bodies. The phenotype becomes much more nuanced in the older child, but the appearance of the nose remains characteristic, and the abdomen remains slightly protuberant. In our study, we analyzed the genotype-phenotype correlation through a literature review that examined phenotypic differences among patients with variants in the three genes associated with 3-M syndrome. Our findings broadened the phenotypic and genotypic spectrum and highlighted the importance of early diagnosis and planned follow-up care, starting in the neonatal period. Accurate phenotyping is extremely important for the diagnostic process: when clinical evaluation is strongly indicative of a specific condition, it can guide diagnostic investigations, and may justify the effort of performing functional studies to confirm the pathogenicity of a variant of uncertain significance.

## Supplementary Information

Below is the link to the electronic supplementary material.

Supplementary Material 1

## Data Availability

The datasets used and analyzed during the current study are available from the corresponding author on reasonable request.

## Abbreviations

ACMG: American College of Medical Genetics
AMP: Association for Molecular Pathology
NGS: Next generation sequencing
OFC: Occipitofrontal circumference
SD: Standard deviation
WG: Weeks of gestation
